# A clinical staging proposal of the disease course over time in non-severe patients with coronavirus disease 2019

**DOI:** 10.1038/s41598-021-90111-y

**Published:** 2021-05-21

**Authors:** Yiting Lin, Yiqun Wu, Ping Zhong, Bingbo Hou, Jielan Liu, Yingying Chen, Jiajun Liu

**Affiliations:** 1 Department of Respiratory and Critical Care Medicine, Xiamen Haicang Hospital, Xiamen, China; 2 Department of Respiratory Section II, The Third Hospital of Xiamen Affiliated to Fujian University of Traditional Chinese Medicine, Xiamen, China; 3 BE and Phase I Clinical Trial Center, The First Affiliated Hospital of Xiamen University, NO.55 Zhenhai Road, Xiamen, 361003 Fujian China; 4 Department of Cardiology, Zhongshan Hospital Affiliated to Xiamen University, Xiamen, China; 5 Department of Internal Medicine, Xiamen Lotus Hospital, Xiamen, China; 6 Department of Cardiac and Cerebral Function, Xiamen Xian Yue Hospital, Xiamen, China; 7 Department of Infectious Diseases, The First Affiliated Hospital of Xiamen University, NO.55 Zhenhai Road, Xiamen, 361003 Fujian China

**Keywords:** Immunology, Diseases, Pathogenesis

## Abstract

Information on the clinical staging of coronavirus disease 2019 (COVID-19) is still limited. This study aimed to propose a clinical staging proposal of the disease course in non-severe patients with COVID-19. In this retrospective study, 108 non-severe patients with COVID-19 were grouped according to the duration from symptoms onset to hospital admission: ≤ 1 week, > 1 to 2 weeks, > 2 to 3 weeks, > 3 to 5 weeks, respectively. The dynamic changes of clinical signs were profiled across the four groups. A clinical staging proposal of the disease course over time was proposed from the perspective of the interaction between the virus and host. The prodromal phase, characterized by pneumonia, significant lymphopenia, and slightly elevated inflammatory markers, occurred in the first week after symptoms onset. In the second week, all the hematological and inflammatory markers were at the peak or bottom. Meanwhile, progressive pneumonia as well as the secondary damage of other organs (e.g. cardiac damage, coagulopathy, etc.) was significant during this period, making the disease progress into the apparent manifestation phase. In the third week, the improvement of the majority of clinical signs accompanied by a relatively high degree of inflammatory response defined the remission phase. After 3 weeks, patients were in the convalescent phase, in which all the indicators were maintained at a relatively normal level. We concluded that the disease course over time in non-severe patients with COVID-19 could be divided into four phases: the prodromal phase (in the first week), the apparent manifestation phase (in the second week), the remission phase (in the third week), and the convalescent phase (after 3 weeks), respectively. In clinical practice, tailored therapies should be considered seriously in different stages of the disease course.

## Introduction

The ongoing coronavirus disease 2019 (COVID-19) pandemic, caused by severe acute respiratory syndrome coronavirus-2 (SARS-Cov-2), continues spreading rapidly worldwide and has impacted health globally on an unprecedented scale. Globally, hundreds of millions of confirmed cases were reported, with total deaths exceeding 2700,000^1^. The clinical spectrum of patients with COVID-19 is quite broad, ranging from mild or no symptoms to severe or critical illness, whereas most reported cases only experienced mild to moderate symptoms^2–4^. However, previous studies have indicated that approximately 10% to 20% of the patients developed severe illness after admission to a hospital^2,5^. In particular, some of them could develop acute respiratory distress syndrome (ARDS), septic shock, acute cardiac injury, acute kidney injury, or even multiple organ failure during hospitalization^3,6,7^. Thus, fully understanding the disease course of COVID-19 plays a crucial role in clinical practice.

Up to now, the disease course of COVID-19 has been noted in several published reports^3,4,6,8^. It was reported that the median times from symptoms onset to dyspnea, sepsis, ARDS, and acute cardiac injury were 7 days, 9 days, 12 days, and 15 days in 191 patients with COVID-19^6^. Of note, early disease progression, manifesting as acute kidney injury, was also observed in the first week after symptoms onset^8^. All these results indicated that COVID-19 patients had different manifestations in different stages of the disease course. However, the disease course of COVID-19 is still poorly explored. Moreover, so far, accumulative data on the current topic were derived from severe or critical patients^3,4,6^, while information on the disease course in non-severe patients with COVID-19 remains scared. In view that more than 80% of COVID-19 patients are non-severe^2^, there is an urgent need to investigate the disease course over time in non-severe patients with COVID-19.

In general, the clinical outcome of COVID-19 is largely determined by virus-host interaction, which was called a “tug-of-war” between SARS-Cov-2 and host antiviral defense^9^. The immune responses, including innate and adaptive immune response and subsequent inflammatory responses, are the major defensive measures in the host antiviral defense. Therefore, the disease course of COVID-19 is due to not only SARS-Cov-2 infection but also the interaction of the virus with the immune system and the subsequent inflammatory responses^10^. Recently, several theoretical stages of the disease course over time have been proposed for COVID-19^11,12^. Nevertheless, these clinical staging proposals are more theoretical. More importantly, no clinical study on the COVID-19 staging has been published to verify these theories so far. Hence, to verify these theories and fully understand the disease course of COVID-19, the purpose of this study was to explore the disease course over time from the perspective of the interaction between the virus and host, with a special focus on proposing a clinical staging which was more applicable to non-severe patients with COVID-19.

## Methods

### Study population and data collection

The study population was consecutive laboratory-confirmed COVID-19 patients admitted to the E3-9 ward in Wuhan Tongji Hospital Guanggu Branch, Huazhong University of Science and Technology between February 10, 2020, and March 26, 2020. This hospital was managed by a multidisciplinary team from Xiamen city during the COVID-19 outbreak. Clinical medical records from non-severe patients with COVID-19 admitted to the hospital were retrospectively analyzed. Based on the “Chinese guideline of diagnosis and treatment for COVID-19” issued by the National Health Commission of People’s republic of China (http://www.nhc.gov.cn/), COVID-19 patients were confirmed by detecting SARS-CoV-2 RNA in pharyngeal swab samples using a virus nucleic acid detection kit in the clinical laboratory of Tongji Hospital. All methods were carried out in accordance with relevant guidelines and regulations (Declaration of Helsinki). This study was approved by the Ethics Committee of Xiamen Haicang Hospital under an expedited review (LW-2020023) and informed consent was waived for this retrospective study.

We collected demographic information, clinical characteristics, laboratory results, and radiological findings from COVID-19 patients on hospital admission within 24 h as previously demonstrated^13^. Clinical characteristics included symptoms onset, the duration from symptoms onset to hospital admission, vital signs at presentation and pre-existing comorbidities. Laboratory results included hematology tests (white blood cell (WBC), lymphocyte count, and hemoglobin, platelet count (PLT)), biochemistry tests (albumin, alanine aminotransferase (ALT), aspartate aminotransferase (AST), urea, creatinine, lactic dehydrogenase (LDH), potassium, sodium, calcium, and chlorine), coagulation tests (prothrombin time (PT), activated partial thromboplastin time (APTT), thrombin time (TT), fibrinogen, and D-dimer), C-reactive protein (CRP), and high sensitive troponin I (hs-cTnI). Radiological findings on hospital admission were recorded according to the degree of lung involvement showed in chest computed tomography (CT). Additionally, as serological tests of SARS-Cov-2 were only applied in patients who were admitted to the hospital in March 2020, the results of serological tests on hospital admission from these patients were collected as well.

According to the “Chinese guideline of diagnosis and treatment for COVID-19”, the severity of COVID-19 was categorized as non-severe, severe, or critical. The severe type was characterized by (1) dyspnea (respiratory frequency ≥ 30 rates per minute); (2) oxygen saturation ≤ 93% in resting state; (3) arterial partial pressure of oxygen (PaO2)/oxygen concentration (FiO2) ≤ 300, and/or lung infiltrates > 50% within 24–48 h (satisfying at least one of the above items)^13^. The critical type was characterized by respiratory failure, shock, or multiple organ dysfunction syndromes. Non-severe patients included patients with mild to moderate pneumonia and satisfied none of the above items. Since the study population was non-severe patients with COVID-19, severe or critical patients were not included in this study. Besides, COVID-19 patients without detailed medical records (e.g., without CT, CRP, etc.) were not included as well. All data were recorded and checked by two physicians (WY and HB) and any discrepancies were resolved by discussion.

### Study design

Recently, three pathological phases of the disease progression over time have been proposed for COVID-19: pre-symptomatic phase (3 days before symptoms onset), mild symptomatic phase (within 1 week after symptoms onset), severe symptomatic phase (during the second week to the third week after symptoms onset), and the recovery phase (3 weeks after symptoms onset)^12^. Moreover, the disease was hypothesized to progress to hyperinflammation phase around 10 days after symptoms onset in patients with COVID-19^11^. Thus, according to the above theories, the enrolled COVID-19 patients were grouped based on the duration from symptoms onset to hospital admission: group 1 (admitted to the hospital within 1 week after symptoms onset); group 2 (admitted to the hospital > 1 week to 2 weeks after symptoms onset); group 3 (admitted to the hospital > 2 weeks to 3 weeks after symptoms onset); group 4 (admitted to the hospital > 3 weeks to 5 weeks after symptoms onset). The duration from symptoms onset to hospital admission was defined as the time from the first symptoms to the hospital admission, and the exact date of the first symptoms had been recorded clearly in the clinical medical records. Demographic, clinical, laboratory, and radiological data were compared across the four groups, and the dynamic changes of clinical signs over time across the disease course were profiled. According to the dynamic profiles and current pathophysiological theory on COVID-19, we sought to propose a clinical staging proposal of the disease course in non-severe patients with COVID-19 from the perspective of the interaction between the virus and host.

### Statistical analysis

The data were analyzed by SPSS statistic 22.0 (SPSS Inc., Chicago, USA). Initially, normality tests were run to assess data distribution using the Shapiro–Wilk test and Q–Q plot. Continuous variables were expressed as the median and interquartile range (IQR) when they were highly skewed distribution, and the differences were analyzed using independent samples Kruskal–Wallis test across the four groups. Categorical values were expressed as frequencies, and the differences were analyzed using χ^2^ test or Fisher’s exact test across the four groups. Besides, a boxplot (without outliers) was drawn to analyze the dynamic change of an indicator if a significant difference for this indicator was found across the four groups. The dynamic changes of the SARS-Cov-2 antibodies were also profiled using a boxplot (without outliers). All statistical significance was defined as *P* < 0.05. However, the *P*-value was adjusted using Bonferroni methods when pairwise comparison in the four groups was performed.

## Results

### Comparisons of baseline demographic and clinical characteristics across the four groups

A total of 108 consecutive non-severe patients with laboratory-confirmed COVID-19 were retrospectively enrolled in the present study. The median age was 57.5 years (IQR 41.5–69.0), and 46.30% of patients (50/108) were over 60 years. Finally, all included patients were discharged from the hospital with a good outcome. The median length of hospital stay was 13.0 days (IQR, 10.0–17.0), ranging from 5 to 37 days. The numbers of the four groups were 27, 28, 27, and 26, respectively. The comparisons of baseline demographic and clinical characteristics across the four groups are shown in Table 1. There were no significant differences in age, sex, comorbidity, medications, and the onset of the symptoms across the four groups.

**Table 1:** Comparisons of baseline demographic and clinical characteristics across the four groups.

Characteristics	Total (*n* = 108)	Group 1 (*n* = 27)	Group 2 (*n* = 28)	Group 3 (*n* = 27)	Group 4 (*n* = 26)	H/χ^2^	*P*
Age (years)	57.5 (41.5–69.0)	54.0 (35.0–69.0)	61.0 (52.5–70.0)	64.0 (41.0–69.0)	50.0 (43.0–65.8)	2.609	0.456
< 60	58 (53.70)	18 (66.67)	12 (42.86)	12 (44.44)	16 (61.54)	4.723	0.193
≥ 60	50 (46.30)	9 (33.33)	16 (57.14)	15 (55.56)	10 (38.46)		
**Sex**							
Male	56 (51.85)	13 (48.15)	16 (57.14)	15 (55.56)	12 (46.15)	0.949	0.814
Female	52 (48.15)	14 (51.85)	12 (42.86)	12 (44.44)	14 (53.85)		
**Comorbidity**							
Hypertension	30 (27.78)	5 (18.52)	9 (32.14)	7 (25.93)	9 (34.62)	2.000	0.255
Diabetes	23 (21.30)	6 (22.22)	3 (10.71)	8 (29.63)	6 (23.08)	3.052	0.384
**Coronary heart disease**							
≥ 1 comorbidity	9 (8.33)	1 (3.70)	3 (10.71)	3 (11.11)	2 (7.69)	1.252	0.741
1–2	39 (36.11)	8 (29.63)	13 (46.42)	9 (33.33)	9 (34.62)	5.344	0.501
≥ 3	16 (14.81)	4 (14.81)	1 (3.57)	6 (22.22)	5 (19.23)		
**Medications**							
1–2	23 (21.30)	2 (7.40)	8 (28.57)	5 (18.52)	9 (34.62)	6.075	0.416
≥ 3	18 (16.67)	6 (22.22)	4 (14.29)	4 (14.81)	7 (26.92)		
**Symptoms of illness onset**							
Fever	59 (54.63)	12 (44.44)	13 (46.43)	18 (66.67)	16 (61.54)	3.969	0.265
Cough	53 (49.07)	11 (40.74)	12 (42.86)	13 (48.15)	17 (65.38)	3.960	0.266

Data are shown as median (interquartile range) or n (%).

*P* values were calculated by Kruskal–Wallis test, χ^2^ test or Fisher’s exact test, as appropriate.

### Comparisons of vital signs, laboratory indices, and radiographic findings across the four groups

The comparisons of vital signs, laboratory indices, and radiographic findings across the four groups are shown in Table 2. Compared with patients in group 1 and group 4, patients in group 2 had a significantly higher level of hs-cTnI, a significantly higher incidence of bilateral pneumonia, and a significantly lower level of SPO_2_ (adjusted *P* < 0.05). Compared with patients in group 4, patients in group 2 had a remarkably higher level of LDH and remarkably lower levels of potassium and lymphocyte count (adjusted *P* < 0.05). Patients in group 4 had a significantly lower level of CRP than those in the other three groups (adjusted *P* < 0.05), while no difference in this level was observed across the other three groups (adjusted *P* > 0.05). Compared with patients in group 4, patients in group 2 and group 3 had significantly higher levels of PT, fibrinogen, and D-dimer and significantly lower levels of albumin and calcium (adjusted *P* < 0.05).

**Table 2:** Comparisons of vital signs, laboratory indices, and radiographic findings across the four groups.

Characteristics	Total (*n* = 108)	Group 1 (*n* = 27)	Group 2 (*n* = 28)	Group 3 (*n* = 27)	Group 4 (*n* = 26)	H/χ^2^	*P*
Temperature (°C)	36.50 (36.33–36.80)	36.60 (36.30–36.80)	36.60 (36.40–37.00)	36.50 (36.30–37.00)	36.50 (36.38–36.63)	3.576	0.311
Heart rate (beats/min)	90.0 (78.3–102.8)	96.0 (83.0–104.0)	89.5 (77.8–96.0)	84.0 (76.0–96.0)	90.0 (81.3–105.3)	3.491	0.322
SBP (mm Hg)	129.0 (118.3–140.0)	126.0 (114.0–135.0)	128.0 (114.5–143.3)	124.0 (116.0–140.0)	135.0 (123.3–150.0)	6.675	0.083
DBP (mm Hg)	80.0 (73.0–89.8)	81.0 (75.0–90.0)	75.5 (71.0–84.8)	78.0 (70.0–90.0)	82.5 (74.8–90.3)	4.990	0.173
SPO_2_ (%)	98.0 (96.0–98.8)	98.0 (97.0–99.0)a	96.5 (94.3–98.0)ac	97.0 (96.0–98.0)	98.0 (97.0–99.0)c	11.642	0.009*
WBC (× 10^9^/L)	5.88 (4.34–7.55)	4.58 (4.02–5.90)de	6.60 (3.60–7.85)	6.45 (4.86–7.75)d	6.13 (5.49–8.03)e	13.049	0.005*
Lymphocyte count (× 10^9^/L)	1.26 (0.84–1.79)	1.17 (0.86–1.66)	1.12 (0.69–1.42)c	1.30 (0.81–1.80)	1.75 (1.26–2.16)c	12.529	0.006*
Hemoglobin (g/L)	129.0 (116.3–139.0)	130.0 (120.0–149.0)	128.0 (120.0–138.5)	129.0 (110.0–138.0)	128.0 (117.0–142.3)	1.813	0.612
PLT (× 10^9^/L)	209.5 (166.8–264.0)	188.0 (156.0–217.0)d	218.0 (147.3–280.3)	256.0 (185.0–360.0)d	201.5 (182.5–248.3)	9.402	0.024*
CRP (mg/L)	10.75 (1.53–49.73)	12.70 (0.90–48.00)e	34.45 (6.63–64.93)c	29.00 (3.90–55.60)f	1.85 (0.50–4.10)cef	19.885	0.000*
Troponin I (pg/mL)	4.90 (1.90–10.53)	1.90 (1.90–8.80)a	10.25 (6.75–15.63)ac	5.30 (1.90–11.7)	1.90 (1.90–3.55)c	23.567	0.000*
Albumin (g/L)	39.00 (33.42–42.85)	40.70 (36.60–43.50)a	34.15 (32.15–40.35)ac	34.80 (30.90–41.40)f	42.55 (39.98–44.63)cf	21.316	0.000*
ALT (U/L)	24.00 (14.00–37.00)	22.00 (13.00–36.00)	24.50 (14.00–34.25)	24.00 (18.00–34.00)	23.50 (13.00–51.25)	0.881	0.830
AST (U/L)	22.50 (16.25–35.50)	26.00 (17.00–33.00)	23.50 (19.00–43.75)	20.00 (14.00–41.00)	20.00 (14.75–30.25)	3.106	0.376
Urea (mmol/L)	4.40 (3.40–5.68)	4.40 (3.10–5.40)	4.60 (3.20–5.65)	4.00 (3.20–5.60)	4.70 (3.68–5.83)	1.821	0.610
Creatinine (μmol/L)	70.00 (55.25–87.75)	64.0 (54.0–83.0)	77.5 (55.0–96.25)	70.0 (57.0–87.00)	69.00 (54.50–83.25)	2.252	0.522
LDH (U/L)	206.50 (169.25–291.25)	204.0 (187.0–246.0)	267.0 (235.0–351.25)c	224.0 (153.0–342.0)	174.00 (157.00–195.75)c	21.496	0.000*
Potassium (mmol/L)	4.07 (3.78–4.32)	3.93 (3.80–4.19)	3.76 (3.49–4.23)c	4.15 (3.98–4.32)	4.21 (4.03–4.47)c	14.211	0.003*
Sodium (mmol/L)	139.10 (136.63–140.68)	138.9 (136.6–140.80)	138.7 (136.03–139.90)	139.2 (136.7–142.40)	139.55 (136.82–140.53)	2.032	0.566
Chlorine (mmol/L)	100.85 (98.23–103.00)	99.70 (97.4–102.9)	99.0 (96.1–102.1)b	102.6 (99.3–104.7)b	100.95 (99.40–102.83)	9.523	0.023*
Calcium (mmol/L)	2.16 (2.07–2.22)	2.16 (2.06–2.21)	2.12 (2.03–2.20)c	2.13 (2.03–2.18)f	2.22 (2.15–2.29)cf	12.471	0.006*
PT (s)	13.70 (13.20–14.10)	13.7 (13.2–14.2)	13.75 (13.23–14.2)c	14.10 (13.5–14.6)f	13.20 (12.88–13.80)cf	16.655	0.001*
APTT (s)	37.70 (36.30–40.90)	38.40 (36.50–43.80)	37.95 (36.73–41.13)	37.8 (35.10–40.60)	37.15 (35.67–40.03)	1.780	0.619
TT (s)	16.50 (15.83–17.30)	16.50 (15.60–17.30)	16.55 (16.05–17.90)	16.70 (15.70–17.70)	16.50 (15.85–17.12)	1.034	0.793
Fibrinogen (g/L)	4.14 (3.11–5.29)	3.98 (3.06–4.94)	5.05 (4.28–6.18)c	4.39 (3.12–6.49)f	3.30 (2.90–3.88)cf	17.392	0.001*
D-dimer (ug/mL)	0.40 (0.22–0.91)	0.35 (0.22–0.64)	0.61 (0.35–1.13)c	0.48 (0.22–1.13)f	0.22 (0.22–0.32)cf	13.195	0.004*
**Chest CT findings**							
Unilateral pneumonia	55 (50.93)	18 (66.67)a	8 (28.57)ac	11 (40.74)	18 (69.23)c	12.882	0.005
Bilateral pneumonia	53 (49.07)	9 (33.33)	20 (71.43)	16 (59.26)	8 (30.77)		

Data are shown as median (interquartile range) or n (%).

*SBP* systolic blood pressure, *DBP* diastolic blood pressure, *SPO*_*2*_ pulse oximeter O_2_ saturation, *WBC* White blood cell, *PLT* platelet count, *CRP* C-reactive protein, *ALT* alanine aminotransferase, *AST* aspartate aminotransferase, *LDH* lactic dehydrogenase, *PT* prothrombin time, *APTT* activated partial thromboplastin time, *TT* thrombin time, *CT* computed tomography.

*P* values were calculated by Kruskal–Wallis test, χ^2^ test or Fisher’s exact test, as appropriate.

*Denoted *P* < 0.05 across the four groups.

a—Denoted adjusted *P* < 0.05 between group 1 and group 2.

b—Denoted adjusted *P* < 0.05 between group 2 and group 3.

c—Denoted adjusted *P* < 0.05 between group 2 and group 4.

d—Denoted adjusted *P* < 0.05 between group 1 and group 3.

e—Denoted adjusted *P* < 0.05 between group 1 and group 4.

f—Denoted adjusted *P* < 0.05 between group 3 and group 4.

### Dynamic profiles of clinical signs over time in non-severe patients with COVID-19

**Figure 1:**
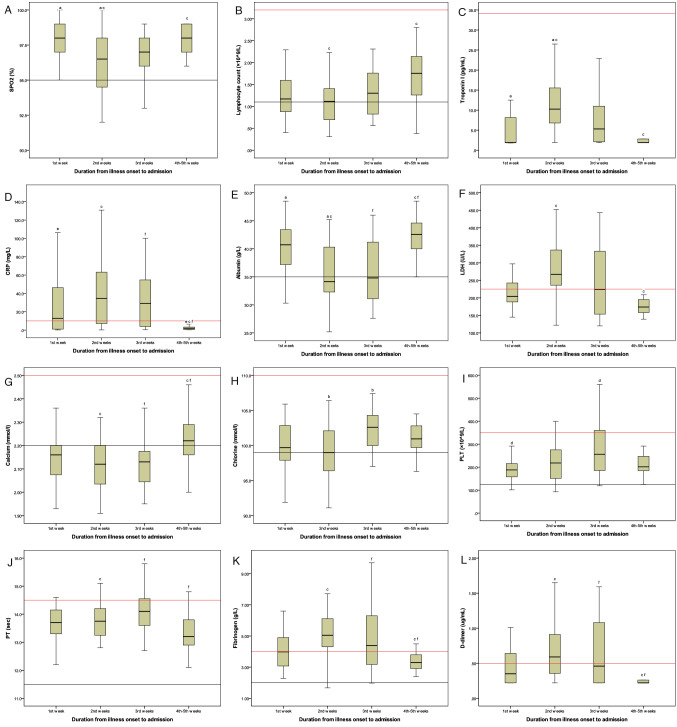
Dynamic profiles of clinical signs over time in non-severe patients with COVID-19. In the first week after symptoms onset, COVID-19 patients showed remarkably decreased levels of lymphocyte count and calcium and slightly increased levels of C-reactive protein, lactic dehydrogenase, fibrinogen, and D-dimer. Meanwhile, the levels of albumin, high sensitive troponin I, and pulse oximeter O_2_ saturation remained unchanged. All the clinical signs, including lymphocyte count, electrolyte, albumin, high sensitive troponin I, C-reactive protein, lactic dehydrogenase, fibrinogen, D-dimer, and pulse oximeter O_2_ saturation, were at the peak (bottom) in the second week after symptoms onset. The majority of clinical signs improved in the third week, whereas C-reactive protein, lactic dehydrogenase, fibrinogen, and D-dimer were still at a relatively high level. All the above indicators were maintained at a significantly low (high) level during the fourth week to the fifth week after symptoms onset. Upper limit of reference interval (red line); Lower limit of reference interval (black line). *SPO*_*2*_ pulse oximeter O_2_ saturation, *PLT* platelet count, *CRP* C-reactive protein, *LDH* lactic dehydrogenase, *PT* prothrombin time.

**Figure 2:**
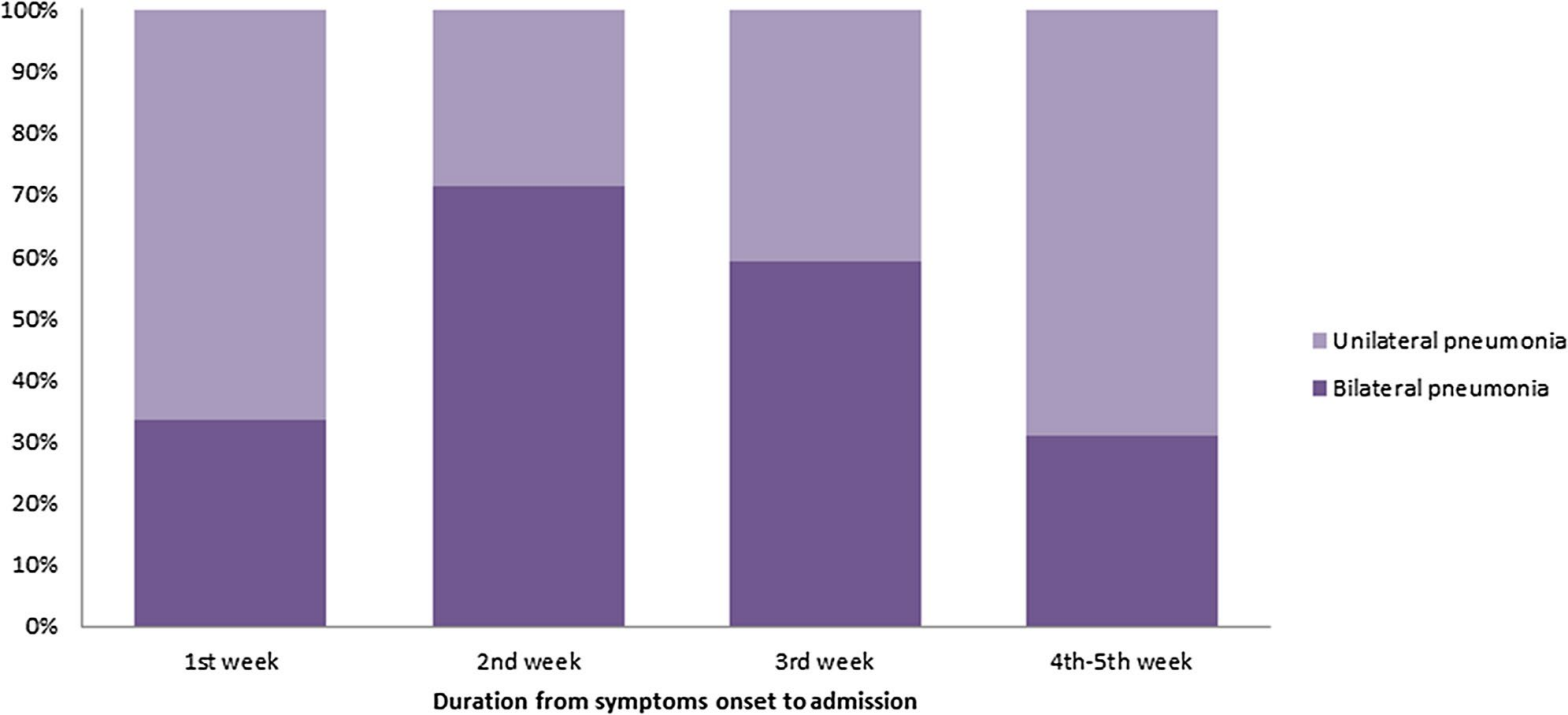
Dynamic profiles of abnormal chest imaging over time in non-severe patients with COVID-19. Pneumonia occurred early in the course of illness and bilateral pneumonia was also observed in the first week after symptoms onset. In the second week after symptoms onset, pneumonia progressed and the highest incidence of bilateral pneumonia was observed during this period. After that, pneumonia improved since the third week after symptoms onset and still had somewhat abnormal chest imaging during the fourth to the fifth week.

The dynamic profiles of clinical signs over time in non-severe patients with COVID-19 are shown in Figs. 1 and 2. In the first week after symptoms onset, COVID-19 patients showed remarkably decreased levels of lymphocyte count and calcium and slightly increased levels of CRP, LDH, fibrinogen, and D-dimer. The levels of albumin, hs-cTnI, and SPO_2_ remained unchanged. Additionally, pneumonia occurred early in the course of illness, and bilateral pneumonia was also observed during this period. All the inflammatory and hematological markers, including lymphocyte count, electrolyte, albumin, hs-cTnI, CRP, LDH, fibrinogen, D-dimer, and SPO_2_, were at the peak (bottom) in the second week after symptoms onset. Meanwhile, the highest incidence of bilateral pneumonia was found during this period. After that, the majority of clinical signs improved in the third week, whereas CRP, LDH, fibrinogen, and D-dimer were still at a relatively high level. All the above indicators were maintained at a relatively normal level despite somewhat abnormal chest imaging during the fourth week to the fifth week after symptoms onset.

### Dynamic profiles of the SARS-Cov-2 antibodies over time in non-severe patients with COVID-19

**Figure 3:**
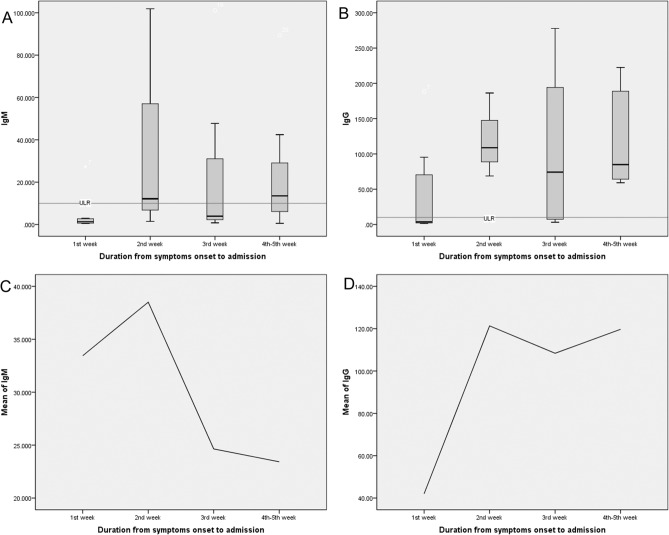
Dynamic profiles of the SARS-Cov-2 antibodies over time in non-severe patients with COVID-19. The tendency of the dynamic changes of the SARS-Cov-2 antibodies was typical. Patients showed remarkably elevated levels of IgM and IgG since the second week after symptoms onset compared with patients who were in the first week after symptoms onset. After that, the relatively high level of IgG was still persistent after 2 weeks, whereas the level of IgM tended to decrease slightly. *ULR* Upper limit of reference interval.

In the present study, a total of 29 patients had a result of serological tests. Among them, there were 11 patients in group 1, three patients in group 2, seven patients in group 3, and eight patients in group 4. Limited by the small sample size of these subgroups, no significant differences in the levels of IgM and IgG were found across the four groups. However, the tendency of the dynamic changes of SARS-Cov-2 antibodies was typical. As shown in Fig. 3, patients showed remarkably elevated levels of IgM and IgG since the second week after symptoms onset. After that, a relatively high level of IgG was still persistent after two weeks, whereas the level of IgM tended to decrease slightly.

### Clinical staging proposal of the disease course over time in non-severe patients with COVID-19

**Figure 4:**
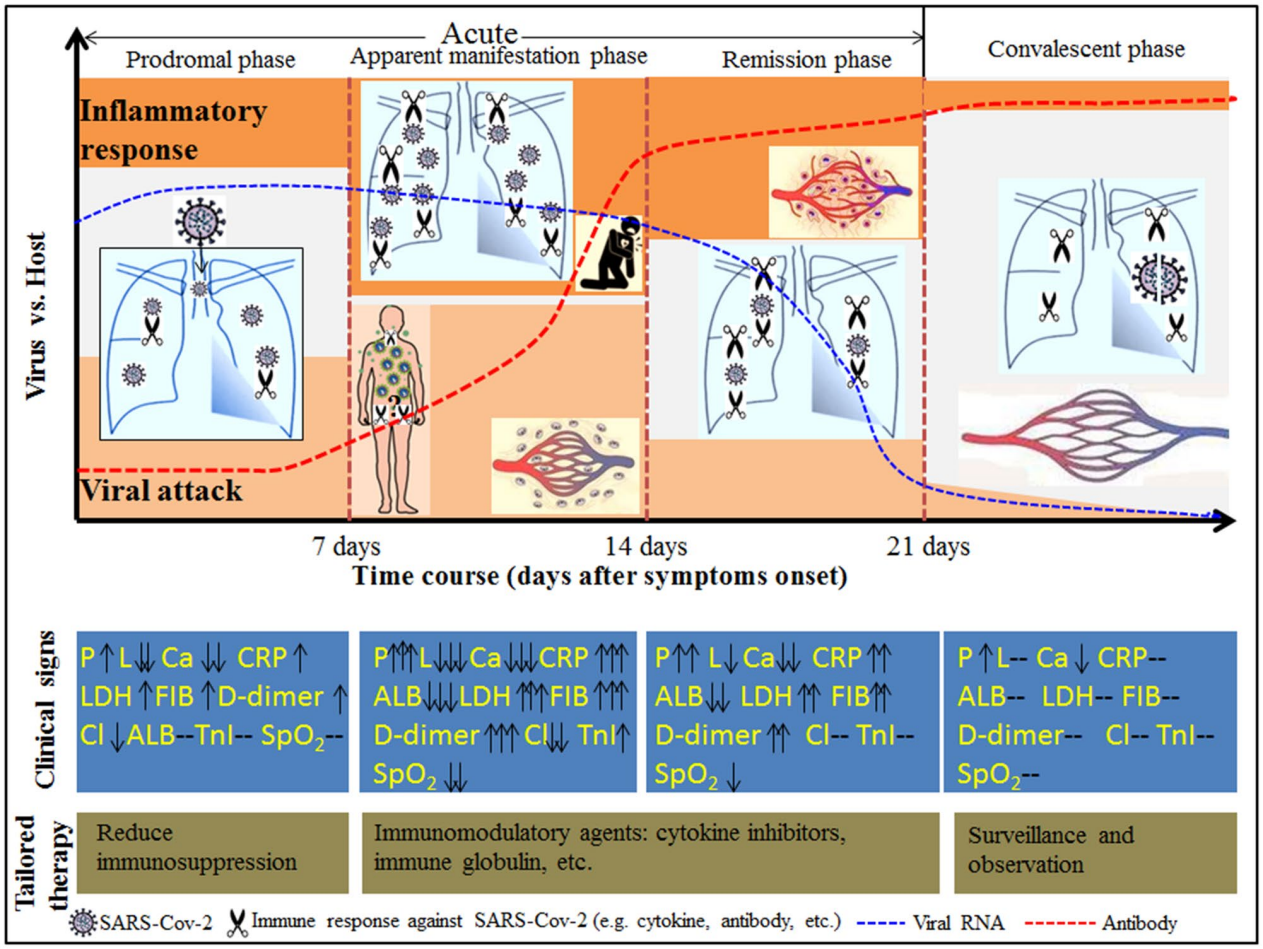
Diagrammatic sketch of the clinical staging proposal of the disease course over time in non-severe patients with COVID-19. The disease course over time in non-severe patients with COVID-19 was divided into four phases: the prodromal phase (in the first week), the apparent manifestation phase (in the second week), the remission phase (in the third week), and the convalescent phase (after 3 weeks). The prodromal phase was characterized by pneumonia, lymphopenia, and slightly elevated inflammatory markers, suggesting that the infection was localized within the lung or the respiratory system. In the second week after symptoms onset, all the hematological and inflammatory markers were at the peak (bottom), indicating that an intense immune response might exist during this period. As a result of the secondary attack and the subsequent systemic inflammatory response, the disease progressed into the apparent manifestation phase. In this phase, pneumonia and other secondary organ damage (e.g. cardiac damage, coagulopathy, etc.) became apparent. After that, due to the production of effective SARS-Cov-2 antibodies, an accelerated viral clearance accompanied by a relatively high degree of inflammatory response defined the remission phase. The first three phases constituted the acute phase of COVID-19. After 3 weeks, COVID-19 patients were in the convalescent phase, in which all the indicators were maintained at a significantly low (high) level despite a positive result of SARS-Cov-2 and somewhat abnormal chest imaging. The dynamic profile of viral RNA was adapted from Lou et al.^27^. *P* pneumonia, *L* lymphocyte count, *Ca* calcium, *Cl* Chlorine, *ALB* albumin, *TnI* high sensitive troponin I, *CRP* C-reactive protein, *LDH* lactic dehydrogenase, *FIB* fibrinogen, *SPO*_*2*_ pulse oximeter O_2_ saturation.

The diagrammatic sketch of the clinical staging proposal of the disease course over time in non-severe patients with COVID-19 is shown in Fig. 4. According to the classical theory of infectious disease, the disease course over time in non-severe patients with COVID-19 was divided into four phases: the prodromal phase (in the first week), the apparent manifestation phase (in the second week), the remission phase (in the third week), and the convalescent phase (after 3 weeks), respectively. During the prodromal phase, the infection was probably localized within the lung or the respiratory system. As the immune response turned white-hot, the disease progressed into the apparent manifestation phase, in which pneumonia and other secondary organ damage (e.g. cardiac damage, coagulopathy, etc.) became apparent. After that, an accelerated viral clearance accompanied by a relatively high degree of inflammatory response defined the remission phase. The first three phases constituted the acute phase of COVID-19, and patients were in the convalescent phase after 3 weeks.

## Discussion

For clinicians, seeing through the clinical signs to perceive the interaction between the virus and host plays a crucial role in fully understanding the pathophysiology of COVID-19. The first step in SARS-Cov-2 infection is the virus binding to a host cell through its target receptor, the angiotensin-converting enzyme 2 (ACE2). Commonly, the virus principally targets airway epithelial cells, alveolar epithelial cells, vascular endothelial cells, and macrophages in the lung, resulting in acute lung injury^10^. Consequently, pneumonia would be a principal manifestation of non-severe patients with COVID-19 who were in the first week after symptoms onset. Moreover, consistent with numerous studies^2–4,6,8^, our results showed that lymphopenia was a prominent feature of SARS-Cov-2 infection in the initial stage. There are multiple mechanisms for lymphopenia of SARS-Cov-2 infection in this stage. On the one hand, T-cell could be directly infected by SARS-Cov-2, causing a cytopathic effect on infected T-cell^14^. On the other hand, a large number of lymphocytes were recruited from the peripheral blood to the site of infection (lung), leading to a decreased level of lymphocyte count in the peripheral blood.

Moreover, it is interesting to note that a remarkable electrolyte imbalance, including hypocalcemia and hypochloremia, was observed in non-severe patients with COVID-19 during this period. First, as ACE2 is known to regulate the renin-angiotensin system (RAS), a reduction in ACE2 function after viral infection could result in a dysfunction of the RAS, which influences fluid/electrolyte balance^10^. Second, it was reported that calcium ions (Ca^2+^) play a pivotal role in membrane entry and fusion of coronavirus^15^, and therefore the entry of SARS-Cov-2 might also lead to a lower calcium concentration^16^. Furthermore, the inflammatory markers such as CRP increased slightly in the first week after symptoms onset, suggesting that the inflammatory response might be caused by a relatively localized infection during this period^11^. Accordingly, the first week after symptoms onset was defined as the prodromal phase, in which the infection might be localized within the lung or the respiratory system. Similarly, this phase corresponds to stage I and stage IIa proposed by Siddiqi et al.^11^ and the mild symptomatic phase proposed by Subbarao et al.^12^.

In the second week after symptoms onset, it was hypothesized that SARS-Cov-2 from the lung could spread through the bloodstream to the tissues (e.g. lung and heart) expressing ACE2^17,18^. The secondary attack was thought to be the major cause of the aggravation of symptoms during this period^17,18^. Thus, we infer that the secondary attack and the subsequent host response both make the disease progress into the systemic effect phase. Interestingly, our results further confirm this hypothesis for the potential pathogenesis of SARS-Cov-2 infection. First, in line with previous studies^19,20^, patients in group 2 had the lowest level of SPO_2_ and the highest incidence of bilateral pneumonia across the four groups, indicating that pneumonia progressed significantly in the second week after symptoms onset. Second, patients in group 2 had a significantly higher level of hs-cTnI compared with patients in either group 1 or group 4, suggesting that cardiac damage of COVID-19 probably occurred in this phase. Similar to our result, cardiac injury, which coincided with progressive pneumonia at 9 days after symptoms onset, was observed in a COVID-19 patient^21^.

Third, in addition to CRP, our results showed that the level of LDH was at the peak in the second week after symptoms onset. As LDH is a marker of the systemic inflammatory response^22^, the peaked levels of CRP and LDH could be considered as the signs of the systemic inflammatory response during this period. Besides, systemic inflammation increases capillary permeability and escape of serum albumin widely, leading to the expansion of interstitial space and increasing the distribution volume of albumin^23^. This is the reason that the level of albumin remained unchanged in the first week but decreased significantly since the second week in this study. Fourth, coagulation abnormalities, including elevated levels of PT, fibrinogen, and D-dimer, were remarkable in this systemic effect phase. This result can be explained by the theory that systemic inflammatory responses, as well as vascular endothelial dysfunction caused by the direct attack of SARS-Cov-2, contributed to the coagulopathy in COVID-19 patients^24^. Retrospectively speaking, it makes sense that coagulation abnormalities were moderate in the prodromal phase due to the localized pulmonary thrombotic microangiopathy^25^.

Fifth, a meta-analysis demonstrated that several inflammatory and hematological markers, including CRP, D-dimer, albumin, and LDH, were useful predictors of the disease severity of COVID-19^26^. Severe patients with COVID-19 were suggested to have higher levels of CRP, D-dimer, and LDH and a lower level of albumin than non-severe patients^26^. In the present study, patients in group 2 had the highest levels of CRP, D-dimer, and LDH but the lowest level of albumin compared with the other three groups. This result suggests that patients who were in the second week after symptoms onset presented the most disease severity of COVID-19. Finally, as we showed, both IgM and IgG raised markedly during this period, indicating that the adaptive immune might be largely activated to contend against the widespread of SARS-Cov-2 within the target organs in this phase. Meanwhile, T- or B cell ‘‘exhaustion’, which is induced by persistence or excessive antigenic stimulation of T and B cells^12^, might be the major contributor to the progressive lymphopenia in this phase. Hence, we defined this systemic effect phase as the apparent manifestation phase, which corresponds to the stage IIb and stage III in Siddiqi et al.’ study^11^ and the first part of the severe symptomatic phase proposed by Subbarao et al.^12^.

In the first two stages, the virus may have gained the upper hand in the “tug-of-war”. However, once the body’s immune system is restored, the balance of victory will slowly lean towards the host in the next stage. In agreement with previous studies^27–30^, one milestone of the host immune response was that the SARS-Cov-2 specific IgG antibody was produced stably in the majority of COVID-19 patients since 14 days after symptoms onset. Moreover, another robust piece of evidence from this study was that lymphopenia improved since the third week after symptoms onset. Steady growth in the level of lymphocyte count was shown to be generally well correlated with neutralization antibody titers in COVID-19 patients^31,32^. Furthermore, it was demonstrated that the viral RNA had fallen off a cliff attributed to the effective SARS-Cov-2 antibodies in the third week after symptoms onset^27^. All these results suggested that the viral clearance might be accelerated when the disease developed into this phase. Meanwhile, a relatively high degree of inflammatory response, manifesting as high levels of CRP, LDH, PT, fibrinogen, and D-dimer and a low level of albumin, was still persistent in this stage. Thus, an accelerated viral clearance accompanied by a relatively high degree of inflammatory response defined the remission phase in the third week after symptoms onset. However, this phase was not mentioned in Siddiqi et al.’ and Subbarao et al.’ theories^11,12^.

After that, the majority of clinical signs maintained at a relatively normal level after 3 weeks, suggesting that the degree of host response almost returned to normal during this period. Moreover, the SARS-Cov-2 specific IgG antibody was also maintained at a significantly high level during this period in this study. Given these results and the data suggested that the viral load in COVID-19 patients gradually decreased over time and had a low level since 21 days after symptoms onset^27,33^, the viral load might also be very low during this period. Similarly, Yu et al.^34^ reported that the viral load of SARS-Cov-2 was found to fluctuate at a low level for more than 9 days before becoming negative. Given this result, some patients may take weeks or even months to achieve complete viral clearance. They may have a long asymptomatic virus-carrying state before the complete viral clearance. Accordingly, we defined this phase as the convalescent phase, which accords with Subbarao et al.’ theory^12^.

In this study, a clinical staging proposal of disease course in non-severe patients with COVID-19 was proposed based on the dynamic profiles of clinical signs. Our findings could help us to fully understand the disease course of COVID-19, providing important implications for clinical management in non-severe patients with COVID-19. On the one hand, as non-severe patients with COVID-19 could develop into the apparent manifestation phase, progressive pneumonia, as well as the secondary damage of other organs (e.g. cardiac damage, coagulopathy, etc.), should be paid special attention in clinical practice. Particularly, more attention should be paid in the patients who are in the second week after symptoms onset. On the other hand, since the immunology underlying clinical signs are different in four stages of the disease, it is necessary to select tailored therapies in the clinical practice of COVID-19. For instance, to reduce immunosuppression, the use of corticosteroids in patients with COVID-19 should be avoided in the early infection phase^11^. Similarly, evidence from the RECOVERY trial showed that dexamethasone may be more harmful than helpful in COVID-19 patients who were in the first week after symptoms onset^35^. Besides, to reduce systemic inflammation, several tailored immunomodulatory agents, such as cytokine inhibitors and immune globulin, were suggested to apply in the apparent manifestation phase^11^.

Despite the intriguing findings of this study, several important limitations should be taken into account. First, since the duration from symptoms onset to hospital admission was relatively long and varied widely in this study, recall bias regarding the date of symptoms onset was great due to the long duration. Given this recall bias, misclassification of disease course might influence the result. Second, the study design was retrospective and not self-controlled in this study. The data of medications used to treat COVID-19 before admission were missing in these patients. Particularly, several important clinical indicators such as viral load, CD4+, and CD8+ were not available owing to the retrospective study design. Thus, the potential imbalance in baseline conditions across the four groups might have an impact on the result, although we have considered several pre-existing conditions including age and comorbidities. Third, owing to the shortage of serological reagents in the early stage of an outbreak, very little data on serological analysis was presented in this study. Hence, the longitudinal changes of SARS-Cov-2 antibodies were unable to be reflected completely in this study. Further studies, which take these variables into account, will need to be undertaken. Fourth, COVID-19 patients could be beneficial from early and multidisciplinary intervention, and thereby the disease course might be shortened in these patients, showing a significantly different staging of COVID-19 with ours. For instance, growing evidence demonstrated that wearing personal protective equipment (PPE), such as a mask and eye protection, could prevent person-to-person transmission of COVID-19^36^. The intervention could reduce viral load and then influence the natural history of COVID-19. Furthermore, metformin was shown to benefit in reducing the mortality rate from SARS-Cov-2 infection^37^, suggesting that the treatments and the status of the pre-existing comorbidities could influence the natural history of COVID-19. Unfortunately, owing to the retrospective design, the detailed medications and the controlled status of the pre-existing comorbidities were not available in the present study. Last but not least, our study is single-centered research with a small sample size of patients per group, and it may be underpowered to detect a significant difference between patients with different disease courses. A longitudinal cohort study with a large number of patients is needed to verify our conclusions in the future. In addition, this study was based on the Chinese population, and validation should be considered when our results are applied to other populations.

## Conclusions

In summary, the disease course over time in non-severe patients with COVID-19 was the result of the interaction between the virus and host. A clinical staging proposal of four immunological phases was proposed in this study: the prodromal phase (in the first week), the apparent manifestation phase (in the second week), the remission phase (in the third week), and the convalescent phase (after 3 weeks), respectively. Therefore, progressive pneumonia as well as the secondary damage of other organs (e.g. cardiac damage, coagulopathy, etc.) should be paid special attention in the clinical management of COVID-19. Particularly, more attention should be paid in the patients who are in the second week after symptoms onset. More importantly, tailored therapies should be considered seriously in different stages of the disease course in patients with COVID-19.

## Data Availability

The datasets used and/or analyzed during the current study are available from the corresponding author on reasonable request.
